# Epidemiological 11-Year Dynamics Study of Acute Myocardial Infarction: A Cohort Study in a Country with a Transitional Healthcare System

**DOI:** 10.5334/gh.1525

**Published:** 2026-02-25

**Authors:** Besfort Kryeziu, Afrim Poniku, Michael Y. Henein, Pranvera Ibrahimi, Arlind Batalli, Edita Pllana, Defrim Morina, Anita Berlajolli, Jehona Krasniqi, Shpend Elezi, Gani Bajraktari

**Affiliations:** 1 Medical Faculty, University of Prishtina, Prishtina, Kosovo; 2 Clinic of Cardiology, University Clinical Centre of Kosova, Prishtina, Kosovo; 3 Imperial College, London, United Kingdom; 4 Siena University, Italy; 5 University of Nicosia, Cyprus; 6 Clinic of Cardiology, University Clinical Centre of Kosova, Prishtina, Kosovo; 7 Department of Public Health and Clinical Medicine, UmeåUniversity, Sweden; 8 Medical Faculty, University of Prishtina, Prishtina, Kosovo; 9 Clinic of Infection Disease, University Clinical Centre of Kosova, Prishtina, Kosovo

**Keywords:** acute myocardial infarction, epidemiology, cardiology, UCCK, Kosovo

## Abstract

**Background and Aim:**

Acute myocardial infarction (AMI) remains a major global health problem, being the leading cause of both morbidity and mortality. We aim to present the temporal trends, demographic, clinical characteristics and risk factors of AMI in Kosovo.

**Methodology:**

We conducted a retrospective, single-center observational study at the Clinic of Cardiology, University Clinical Center of Kosovo, having analyzed all patients admitted with AMI between January 2014 and December 2024. STEMI and NSTEMI cases were diagnosed according to ESC criteria. Patient’s risk factors, biomarkers, PCI, and outcome data were extracted from hospital clinical records. Latent class analysis identified patient subgroups based on risk profiles. Temporal trends and projections of AMI incidence (per 100,000 population) were analyzed using polynomial and Joinpoint regression models. Statistical comparisons employed Chi-squared, t-tests, or Mann-Whitney U tests.

**Results:**

Over the course of 11 years, 13,099 AMI patients (mean age 63.8 years; 29% female) were admitted; 55% had STEMI and 45% NSTEMI. Annual Age-standardized incidence increased from 23.5 to 86.4 per 100,000 (2014–2021) then fell to 71.3 in 2024. Hypertension (66%), smoking (47%), diabetes (34%) and dyslipidemia were highly prevalent. Latent class analysis identified four distinct patient clusters with varying combinations of smoking, diabetes, hypertension and family history of cardiovascular disease (CVD) (p < 0.001). STEMI patients were younger, more often male and smokers, while NSTEMI patients were older with higher rates of diabetes, hypertension and prior LBBB. In-hospital mortality was 9.15%, higher for STEMI (~12%) than NSTEMI (~6%), and declined markedly over time (19.3% in 2014 vs 7–10% in 2022, p < 0.001).

**Conclusions:**

In a developing country, Kosovo, STEMI was more frequent than NSTEMI, affecting younger male patients. The leading risk factors included arterial hypertension, smoking, diabetes mellitus, and a family history of CVD. The decline in acute MI related mortality over recent years, can be explained by the increasing use of myocardial reperfusion procedures. Furthermore, the rates of acute MI related complications are not different from neighboring countries.

## Graphical Abstract

**Figure:**
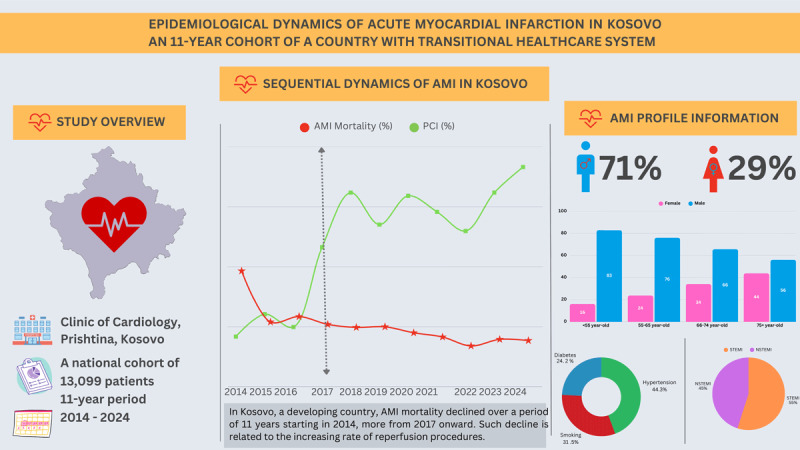


## Introduction

Acute myocardial infarction (AMI) remains a major global health problem, being the leading cause of both morbidity and mortality (1,2). In 2019, approximately 17.9 million individuals died from cardiovascular diseases (CVD), accounting for 32% of all global deaths. Of those deaths, 85% were attributable to MI and cerebrovascular accident (3). During 2021, CVD accounted for 40.3% of total deaths in the USA (4). Likewise in Europe, the respective CVDs accounted for 42.5% of all annual deaths, equivalent to 10,000 deaths every day (5,6). Despite that, a significant decline in CVD and MI related mortality has recently been seen, thanks to the current preventive measures, routine screening, early diagnosis, advanced treatments and improvement of healthcare access. However, disparities between different parts of the world have been identified, with over 75% of CVD deaths occurring in low- and middle-income countries (3,7). Southeastern Europe experiences twice as high death rates from ischemic heart disease in individuals under 65 years of age compared to Western Europe (8). Trends of outcomes of acute MI, insights into the modifiable risk factors, CV prevention and health system planning remain to be established. Kosovo is an upper-middle-income country (UMIC), with a healthcare system that has undergone many reforms towards modernization, aiming at implementing proven successful strategies (9). The country has had significant CV health problems compared to Western Europe (10,11,12). The University Clinical Centre of Kosovo (UCCK), a tertiary healthcare center, is the only public hospital in Kosovo which started primary PCI for acute MI in 2014 (13). By 2018, UCCK had expanded to provide 24/7 primary percutaneous coronary intervention (PPCI) coverage for the entire country, significantly improving timely reperfusion treatment for ST-elevation MI (STEMI) patients (13). With a current lack of integrated digitalized healthcare system, it is challenging to provide robust clinical data on specific factors that might impact overall cardiac care of such patients. Thus, in addition to addressing the existing knowledge gap, the objective of this study is to present the incidence, demographic and clinical characteristics, risk factors and temporal trends of AMI in Kosovo. The study also provides insights into evidence-based preventive measures, healthcare policymaking and planning for the foreseen optimum CV management.

## Methods

### Study design and settings

We conducted a retrospective, single-center hospital-based observational cohort study, analyzing the epidemiological data on clinical characteristics, temporal trends and treatment outcome of patients admitted with AMI in Kosovo over 11-year period, starting in 2014. The study was conducted at the Clinic of Cardiology, UCCK, the national center for advanced CV care.

### Population and data sources

The study population consisted of all patients hospitalized at Clinic of Cardiology, UCCK with confirmed diagnosis of AMI, based on clinical presentation, ECG findings and cardiac enzymes assessment, between 1 January 2014 and 31 December 2024. The population cohort consisted of all patients with AMI from the entire country, with both STEMI and non-ST-segment elevation myocardial infarction (NSTEMI) according to the ESC diagnostic criteria (14). Demographics, biochemical and cardiac markers, comorbidities, risk factors, hospitalization data, detailed PCI procedural information, left ventricular ejection fraction (LVEF) and patient’s clinical outcome data were retrospectively collected from clinical records archived at the hospital, including patient admission and discharge charts. This was performed using standardized internal case-identification criteria that relied on clinical documentation of the AMI diagnosis, the standard electrocardiographic findings, and elevated myocardial biomarkers aligned with the internationally established diagnostic criteria for AMI. For standardized diagnostic case identification, AMI hospitalizations were identified using ICD-10 diagnosis codes. We cross-checked these codes with clinical documentation, ECG findings, and cardiac biomarker results to confirm alignment with ESC diagnostic criteria. We performed continuous data quality checks and manual validation to ensure data completeness and cohesion (15,16).

### Variables and definitions

Demographic variables included age (in years) and sex, recorded at the time of patient admission. CV risk factors and comorbidities obtained from the patient medical history included smoking status, diabetes mellitus (DM), hypertension, dyslipidemia, COPD (chronic obstructive pulmonary disease) and a family history of CVD. Previous relevant medical history/events, including MI or stroke, were also documented. STEMI and NSTEMI diagnosis was stratified according to conventional ECG criteria and cardiac biomarkers levels. Clinical variables included cardiogenic shock and left bundle branch block (LBBB) at admission, as well as laboratory (glucose, lipid profile, urea, creatinine and full hematology) and cardiac biomarkers (creatine kinase-MB and troponins) results, during hospitalization. Data on patient hospitalization outcome and data on coronary angiography and primary PCI, in-hospital mortality and referral rates to other centers were also collected. The primary outcome of the study was incidence of AMI, overall and stratified as STEMI and NSTEMI. Incidence was defined as the number of new AMI cases admitted to UCCK between 2014 and 2024, expressed per 100,000 population. Secondary outcomes included risk factors cluster distribution.

### Statistical analysis

Descriptive statistical analysis was conducted to summarize patient demographics, risk factors, clinical and biochemical parameters, treatments, and hospitalization outcomes over the study period. All continuous variables were presented as means and standard deviations (SD) or medians with interquartile ranges (IQR), depending on the data distribution. All categorical variables were expressed as frequencies and percentages. We performed latent class analysis (LCA) using four variables; smoking, diabetes mellitus, hypertension and family history of CVD, to identify unobserved subgroups of patients with similar CV risk profiles. Model classes and model fit was evaluated using the Bayesian Information Criterion (BIC), Akaike Information Criterion (AIC), and interpretability of class structure, with lower values indicating improved fit, alongside evaluation of clinical interpretability and stability of the resulting class structures. Accordingly, a 4-class model was selected based on optimal fit and clinical coherence. Each patient was assigned to a class, based on maximum posterior probability of the observed patterns, such as smoker and family history of CVD (Class 1); DM and smoker (Class 2); DM and hypertension (Class 3); and family history of CVD and DM (Class 4). Trend differences between groups were expressed using Pearson’s Chi-square test for categorical variables and *t*-test or Mann-Whitney U test for continuous variables. Timely trends in AMI cases were assessed using a second-degree polynomial regression model to capture potential non-linear patterns. Joinpoint regression was then applied to detect significant inflection points in the trend using segmented linear modeling. Forecasting of AMI incidence per 100,000 population for 2025–2027 was performed based on the polynomial model, including 95% confidence intervals which were calculated as model-based confidence intervals for the estimated mean trend. The population denominator (1,782,115) was based on official data from 2014 census from Kosovo Agency of Statistics. To minimize the chance that results were due to coincidence, all tests were two-tailed, with p-values < 0.05 being considered as statistically significant. All statistical analyses were performed using R (version 4.1.1).

### Ethical considerations

This study was conducted complying with the principles of the Declaration of Helsinki as well as applicable laws for data protection and ethical research practices within Kosovo. Ethical approval was obtained from the Ethical Committee of Medical Faculty University of Prishtina (Protocol number 1500, date 21/02/2025) before data collection and analysis. All patients’ records were anonymized and coded appropriately, hence there was no need to obtain written informed consent.

## Results

### Baseline characteristics and epidemiological profile of the study group

**Figure 1:**
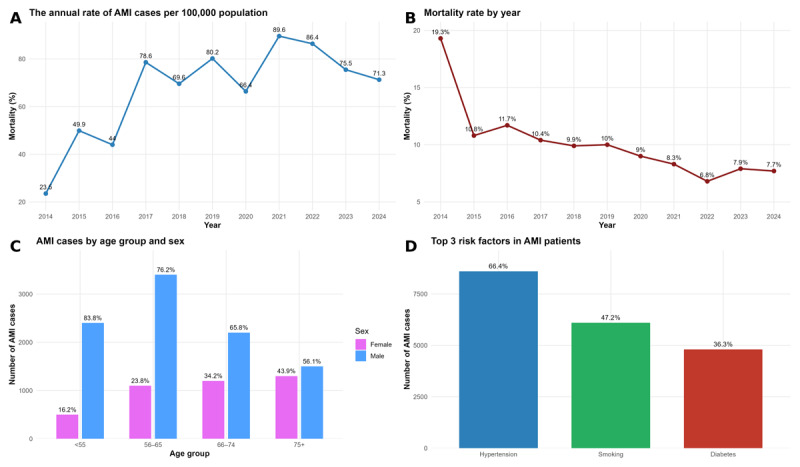
Epidemiological trends of morbidity and mortality, age-sex distribution and main risk factors of patients hospitalized with acute myocardial infarction in Clinic of Cardiology, UCCK, Kosovo, 2014–2024.

From 2014 to 2024, a total of 13,099 patients with AMI were hospitalized in the Clinic of Cardiology, UCCK. Overall, 7,156 (55%) were diagnosed with STEMI, and 5,943 (45%) with NSTEMI, the difference in the incidence between the two groups was significant p < 0.001. Total patients’ mean age was 63.8 ± 11.5 years, and 71% were males. An increasing trend of AMI admissions at our clinic was observed during the study period, from 418 cases in 2014 to 1,271 cases in 2024, with a peak of 1,597 cases in 2021. These results showed a statistically significant fluctuation during 11-year trend (p < 0.001). Among the study population, 47% were active smokers and 34% had type 2 DM. Hypertension remained the most prevalent comorbidity, affecting 66% of patients. A family history of CVD was reported in 42% of patients. COPD was less frequent, present in only 3.3% of cases (p < 0.001 for all). The prevalence of smoking declined over the years, but in contrast DM showed a mild increasing trend. The mean duration of patient’s hospitalization was 5.0 ± 4.4 days. Cardiogenic shock occurred in 4.5% of patients, and LBBB was present in 3.3%. The mean LVEF was 50.2 ± 9.0%, with statistically significant annual variation (p < 0.001). Coronary angiography was performed in 8,677 (73%) patients, and primary PCI in 6,595 (56%) patients. Initially, 9.8% of patients were referred to other institutions (mostly private), but this rate fell from the highest 36% (2016) to recently 0% during 2021–2022. The mean referral rate to other institutions in 2023 and 2024 was 3.5%. Both, primary PCI and coronary angiography showed a significant steep increase over the years (p < 0.001). The overall mean duration of patient’s hospitalization was 5.3 ± 4.3 days, whilst for STEMI patients it was 4.8 ± 4.1 days. The overall in-hospital mortality rate was 9.15%; the highest was 19.3% in 2014 and the lowest was 6.8% in 2022, showing a significant drop over the years (p < 0.001) (Table 1, Figure 1).

**Table 1:** Baseline characteristics, clinical presentation, management and outcomes of patients hospitalized with acute myocardial infarction in Clinic of Cardiology, UCCK, Kosovo, 2014–2024.


VARIABLE	OVERALL n = 13,099^1^	2014 n = 418^1^	2015 n = 890^1^	2016 n = 785^1^	2017 n = 1,400^1^	2018 n = 1,241^1^	2019 n = 1,429^1^	2020 n = 1,184^1^	2021 n = 1,597^1^	2022 n = 1,539^1^	2023 n = 1,345^1^	2024 n = 1,271^1^	p-VALUE^2^

**AMI classification**													<0.001

NSTEMI	5,943 (45%)	144 (34%)	271 (30%)	278 (35%)	542 (39%)	498 (40%)	799 (56%)	455 (38%)	667 (42%)	768 (50%)	864 (64%)	657 (52%)	

STEMI	7,156 (55%)	274 (66%)	619 (70%)	507 (65%)	858 (61%)	743 (60%)	630 (44%)	729 (62%)	930 (58%)	771 (50%)	481 (36%)	614 (48%)	

**Mean age**	63.8 ± 11.5	62.5 ± 12.7	62.5 ± 11.0	62.4 ± 12.3	62.9 ± 11.3	63.5 ± 11.6	64.4 ± 11.9	62.9 ± 11.5	63.8 ± 11.0	64.5 ± 11.1	65.2 ± 11.6	64.8 ± 11.3	<0.001

**Gender**													<0.001

Female	3,759 (29%)	138 (33%)	268 (30%)	228 (29%)	417 (30%)	361 (29%)	419 (29%)	275 (23%)	442 (28%)	440 (29%)	422 (31%)	349 (27%)	

Male	9,340 (71%)	280 (67%)	622 (70%)	557 (71%)	983 (70%)	880 (71%)	1,010 (71%)	909 (77%)	1,155 (72%)	1,099 (71%)	923 (69%)	922 (73%)	

**Smoking**	6,185 (48%)	235 (56%)	513 (58%)	417 (53%)	698 (50%)	655 (53%)	704 (49%)	572 (48%)	697 (45%)	583 (46%)	579 (43%)	532 (42%)	<0.001

**Diabetes mellitus**	4,759 (36%)	131 (31%)	301 (34%)	285 (36%)	409 (29%)	445 (36%)	575 (40%)	445 (38%)	581 (36%)	561 (36%)	510 (38%)	516 (41%)	<0.001

**Hypertension**	8,697 (66%)	209 (50%)	515 (58%)	463 (59%)	904 (65%)	821 (66%)	919 (64%)	993 (84%)	1,051 (66%)	1,043 (68%)	913 (68%)	866 (68%)	<0.001

**Positive anamnesis for CVD**	5,207 (41%)	129 (31%)	309 (35%)	232 (30%)	517 (37%)	457 (37%)	608 (43%)	544 (46%)	669 (43%)	594 (46%)	581 (43%)	567 (45%)	<0.001

**COPD**	431 (3.5%)	27 (6.6%)	37 (4.2%)	21 (2.7%)	31 (3.3%)	57 (5.0%)	67 (4.7%)	37 (3.1%)	36 (2.3%)	30 (1.9%)	50 (4.0%)	38 (3.0%)	<0.001

**Hospitalization duration**	5.0 ± 4.4	4.7 ± 4.9	3.4 ± 3.9	3.5 ± 4.2	4.4 ± 4.1	5.4 ± 3.4	5.3 ± 3.7	5.1 ± 3.1	5.6 ± 4.8	6.1 ± 5.1	5.4 ± 5.4	4.8 ± 4.3	<0.001

**Cardiogenic shock**	593 (4.5%)	7 (1.7%)	7 (0.8%)	10 (1.3%)	69 (4.9%)	32 (2.6%)	87 (6.1%)	61 (5.2%)	82 (5.1%)	83 (5.4%)	80 (5.9%)	75 (5.9%)	<0.001

**LBBB**	434 (3.4%)	10 (2.4%)	26 (2.9%)	10 (1.3%)	31 (2.2%)	26 (2.1%)	140 (9.8%)	27 (2.3%)	33 (2.2%)	46 (3.6%)	39 (2.9%)	46 (3.6%)	<0.001

**Ejection fraction (%)**	50.2 ± 9.0	52.4 ± 11.9	52.6 ± 9.7	51.5 ± 10.1	49.6 ± 10.0	50.5 ± 8.5	49.7 ± 8.9	50.6 ± 7.9	50.0 ± 8.7	48.6 ± 8.9	50.4 ± 8.2	50.3 ± 9.2	<0.001

Coronarography performed*	*8,677 (73%)*	*86 (28%)*	*343 (48%)*	*230 (45%)*	*586 (67%)*	*997 (83%)*	*981 (70%)*	*923 (80%)*	*1,282 (80%)*	*1,148 (75%)*	*1,054 (81%)*	*1,047 (86%)*	<0.001

PPCI performed*	*6,595 (56%)*	*62 (20%)*	*190 (27%)*	*117 (23%)*	*419 (48%)*	*788 (65%)*	*771 (55%)*	*740 (64%)*	*942 (59%)*	*823 (53%)*	*855 (65%)*	*888 (73%)*	<0.001

In-hospital mortality*	*1,081 (9.15%)*	*59 (19.22%)*	*77 (10.77%)*	*59 (11.66%)*	*90 (10.36%)*	*120 (9.94%)*	*139 (9.96%)*	*104 (8.97%)*	*132 (8.27%)*	*104 (6.76%)*	*103 (7.87%)*	*94 (7.73%)*	<0.001

**Referred outside UCCK**	1,279 (9.8%)	111 (27%)	175 (20%)	279 (36%)	531 (38%)	34 (2.7%)	34 (2.4%)	24 (2.0%)	0 (0%)	0 (0%)	36 (2.7%)	55 (4.3%)	<0.001


^1^n (%); Mean ± SD. ^2^Pearson’s Chi-squared test; Kruskal-Wallis rank sum test.

### CV risk factors profile in the study population

**Figure 2:**
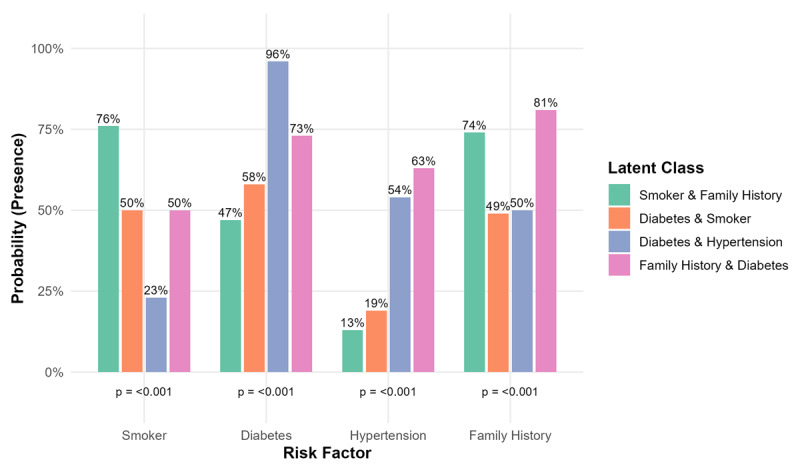
Latent class-derived risk factor profiles among patients hospitalized with acute myocardial infarction in Clinic of Cardiology, UCCK, Kosovo, 2014–2024.

Latent class analysis identified four distinct subgroups of AMI patients based on the presence of key CV risk factors, including smoking, DM, hypertension, and positive family history of CVD. Class 1 exhibited the highest prevalence of smoking (76%) and a notable prevalence of family history of CVD (74%) and DM (47%) while rates of hypertension (13%) were comparatively lower. Conversely, the Class 2 had substantial levels of both DM (58%) and smoking (50%) and family history of CVD (49%), with moderate representation of hypertension (19%). The Class 3 was characterized by a very high prevalence of DM (96%), hypertension (54%) and family history of CVD (50%), with the lowest rate of smoking (23%). Class 4 showed the highest proportion of family history of CVD (81%), a high rate of DM (73%), moderate rate of hypertension (63%) and lower smoking prevalence (50%). Across all four classes, the proportions of each risk factor differed significantly (p < 0.001 for all comparisons), indicating significant heterogeneity in risk factor clustering among AMI patients (Figure 2).

### Eleven-year trend analysis and dynamics of AMI epidemiological burden

**Figure 3:**
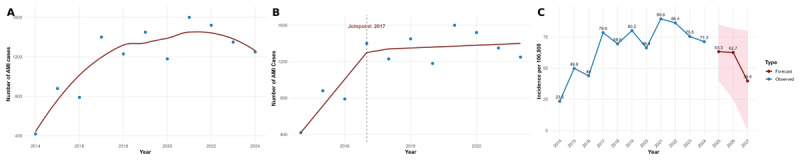
Eleven-year trend analysis and dynamics of AMI in Kosovo from 2014 to 2027; **(A)** Polynomial trend analysis of the number of AMI cases shows a rising pattern up to around 2021, followed by a decline; **(B)** Joinpoint regression analysis identified a statistically significant inflection point in 2017, where the increasing trend in case numbers plateaued; **(C)** Incidence per 100,000 population forecasting for the years 2025–2027 shows a continued decline, with 95% confidence intervals (shaded area).

A second-degree polynomial regression model revealed a nonlinear trajectory in the number of AMI cases from 2014 to 2024. The number of cases increased from 454 in 2014 to a peak of 1,594 in 2021, followed by a declining trend toward 1,254 cases in 2024. A single joinpoint was detected in 2017, indicating a statistically significant change in trend slope, using segmented regression. From 2014 to 2017, AMI cases increased sharply, but from 2017 onward, the trend stabilized with a more modest increase or plateau. Segmented regression identified a statistically significant joinpoint in 2017, indicating a change in the annual trend of AMI cases. Before 2017, the number of AMI cases increased significantly at an annual rate of +325 cases (p < 0.001). After 2017, the increase flattened with a non-significant slope of +21 cases annually (p = 0.61), suggesting stabilization in AMI cases. When standardized by population (incidence per 100,000) was tested, AMI rates proved to have risen from 23.5 per 100,000 in 2014 to a peak of 89.6 per 100,000 in 2021, followed by a decline to 71.3 per 100,000 in 2024. Applying the retrospective data, we performed future forecasting analysis using the polynomial model. The results projected a continued decline in incidence, with rates estimated at 63.3 per 100,000 for 2025, 52.7 per 100,000 for 2026, and 39.8 per 100,000 in 2027. The forecast 95% confidence interval presented as a shaded pink area, indicates uncertainty around the projections but supporting the overall declining trend (Figure 3).

## Discussion

To our knowledge and to date, this decade-long extended study is the first to analyse epidemiological time trends of acute MI in Kosovo. It shows that the number of cases rose sharply until 2017, then fell after 2018. It also shows that invasive treatment has significantly improved clinical outcomes and mortality has fallen. Despite the positive overall management progress, the majority of patients with AMI continue to be younger males who have many modifiable risk factors among other medical chronic conditions. The consistent fall in the number of cases from 2018 onwards reflects a significant change in the approach of managing AMI patients. Such decline in the incidence of AMI observed is consistent to that reported in a population-based registry from Kaunas, Lithuania, among middle-age adults between 2000–2023, with similar patterns in men and women (17). An annual decline in the incidence of AMI has also been reported in Norway from 2013–2021 (18), particularly among native Norwegians, compared to immigrants from former Yugoslavia (19). In Western and Northern Europe, long-term declines in AMI rates and short-term mortality have been attributed to improved primary prevention and risk-factor control (e.g., smoking reduction, better control of dyslipidemia and blood pressure), wider use of guideline-directed therapies, and faster access to reperfusion and organized systems of acute cardiac care (20). These findings reflect a similar downward trend to what we observed in Kosovo after 2017, particularly after expanding PPCI coverage to become a 24/7 provision for the entire country. The Kosovo AMI transient spike in 2021, we identified, may be linked to the COVID-19 pandemic. Indeed, during COVID-19 lockdown, there was marked decline in the number of hospital admissions and underreporting of patients with AMI worldwide, reflecting the rebound effect of transient spike in AMI cases afterward (21,22).

With regards to conventional atherosclerosis risk factors and their impact on the results, this study showed a high prevalence of smoking, DM and hypertension among patients in Kosovo. These findings are compatible with the Heart of the World study which showed that approximately 80% of CVDs occurred in low- and middle-income countries, where Central Europe, Eastern Europe, and Central Asia regions face the highest levels of CVD mortality. Also, similar to our findings, the Heart of the World study highlights the impact of uncontrolled blood pressure, being the worst global risk factor, despite large regional disparities in mortality, prevention policy implementation and health-system investments (23). Furthermore, in a prospective cohort of 156,424 participants across 17 countries of differing income levels, CV-risk-factor burden (measured by the INTERHEART Risk Score) was highest in high-income countries, but the incidence of major CV events and related mortality was substantially higher in low- and middle-income countries, consistent with disparities in prevention and treatment access (24). These findings highlight the importance of risk factors control in reducing the burden and improving clinical outcome of AMI. It must be mentioned that despite the rough transitional phase of Kosovo’s healthcare, our study shows a remarkable progress in the national acute coronary care over the past decade. The proportion of patients undergoing coronary angiography doubled and primary PCI performed in over half of them. The establishment of enhanced coronary angiography and PPCI services in Kosovo, has markedly reduced the overall AMI related complications. These findings match those from Denmark which showed a significant fall in one-year risk of major CV events (25). Despite these gains, Kosovo remains facing specific challenges including longer hospital stay, referral to private institutions, missing post-discharge and long-term follow-up, secondary prevention, etc. Overall, our study has several strengths. We used data from a large national cohort, with details on demographics, risk factors, treatments, outcomes, and advanced temporal analyses including forecasting. In addition, we used the same internationally-approved AMI definitions and treatment protocols for the whole cohort in order to justify comparison with other national published results within and outside the region. Our study also has certain limitations that must be acknowledged. Firstly, the retrospective design and reliance on hospital records may lead to misclassification or missing data on comorbidities, accurate timing of symptom onset and procedures undertaken. Secondly, the study includes only hospitalized AMI cases, excluding out-of-hospital deaths or undiagnosed MIs, so the incidence we reported may be underestimated. Thirdly, data on long-term follow-up after hospital discharge was not available, hence precluding assessment of recurrent events or post-discharge mortality. Due to Kosovo yet lacking a fully integrated national digital health system, routine assessment of performance indicators along the AMI care pathway has been limited. For this reason, establishing a global, real-time CV registry has become a priority for future monitoring of healthcare quality in Kosovo.

## Conclusions

In a developing country, Kosovo, STEMI was more frequent NSTEMI, affecting young males, while NSTEMI more frequently affected old females. The underlying leading risk factors proved to be arterial hypertension, smoking, DM, and a family history of CVD. The decline in acute MI related mortality over recent years, can be explained by the increasing myocardial reperfusion procedures. Compared with neighboring countries, the rates of acute MI related complications are not different. Thus, a healthcare system integrating primary care services, referral hospitals, and tertiary care centers represents a national priority, as it would support ongoing monitoring of service delivery and patient outcomes and improve coordination across levels of care, with particular emphasis on prevention and management of CV disease.
